# Phylogenetic Analysis of Small Hive Beetles From Native to Introduced Populations

**DOI:** 10.3389/fgene.2022.900795

**Published:** 2022-05-19

**Authors:** Wen Feng Bai, Junfeng Liu, Yuanzhen Liu, Wensu Han, Jay D. Evans, Qiang Huang

**Affiliations:** ^1^ Honeybee Research Institute, Jiangxi Agricultural University, Nanchang, China; ^2^ Jiangxi Province Key Laboratory of Honeybee Biology and Beekeeping, Jiangxi Agricultural University, Nanchang, China; ^3^ Periodicals Agency, Jiangxi Agricultural University, Nanchang, China; ^4^ Department of Medical Biochemistry and Microbiology, Science for Life Laboratory, Uppsala University, Uppsala, Sweden; ^5^ Environment and Plant Protection Institute, Chinese Academy of Tropical Agricultural Sciences, Haikou, China; ^6^ USDA-ARS Bee Research Laboratory, Beltsville, MD, United States

**Keywords:** small hive beetle, phylogeny, SNP, evolution, honeybee

## Abstract

The small hive beetle (SHB), a social parasite of beehives, is native to sub-Saharan Africa and has spread to America, Europe, and Australia. Recently, these beetles invaded China, causing widespread colony collapses in the honeybee, *Apis cerana*. In this study, single nucleotide polymorphisms (SNPs) were identified in the beetle genome from its native range (Africa), a region that was invaded by SHBs nearly 30 years ago (America), and more recent invasions (Asia). The beetles in the United States formed the earliest branch and show signs of two decades of gene flow and local adaptation to differentiate this population from the native ones. The beetles in China were deep branched and showed the highest fixation index when compared to the US populations. The number of SNPs in overexpressed genes was significantly higher than the transcriptome. Gene-expression profiles presented here distinguish the characters between adult and larvae SHBs.

## Introduction

The small hive beetle (*Aethina tumida* Murray, hereafter SHB) is a parasite of bee colonies. This beetle belongs to the family Nitidulidae; members of this family mainly feed on decaying vegetables, ripened fruit, and sap (Mckenna et al., 2015; Neumann et al., 2016). SHBs can survive on a wide range of food but thrive in the beehive that provides shelter and protein-rich food (Cuthbertson et al., 2013; Neumann et al., 2016). The adult beetles are attracted by beehive volatiles and invade the hive around dusk (Torto et al., 2005; Torto et al., 2007; Graham et al., 2011). Once inside the beehive, adult beetles employ a “sit and wait” strategy until an opportunity for reproduction arises (Neumann et al., 2015). The adult beetles lay eggs in cracks, which hatch in approximately 72 h (Neumann et al., 2016). Larvae are the most damaging stage for the beehive as they tunnel through the combs and ferment the honey, which attracts other SHBs (Benda et al., 2008). The larvae can feed on pollen, honey, and bee brood, which is also a vector of bee viruses (Eyer et al., 2009; Huang et al., 2019; Huwiler et al., 2020). At the later stage, the larvae crawl out of the beehive and pupate in soil, which may take up to months depending on the temperature and humidity (Neumann et al., 2016; Cornelissen et al., 2020). Once adult SHBs emerge from the soil, they search for and fly to beehives individually or in swarms (Neumann and Elzen, 2004).

The SHB is endemic in sub-Saharan Africa, and the damage to local honeybees is minor, as the native bees can better guard the entrance and the comb (Neumann et al., 2016). Outside the native range, SHBs were first reported in the United States in 1996 and caused considerable damage to apiculture (Hood, 2000; van Engelsdorp et al., 2007). SHBs were then reported in Canada and Australia in 2002 (Gillespie et al., 2003; Clay, 2006), Mexico in 2007 (Del Valle Molina, 2007), Italy and the Philippines in 2014 (Palmeri et al., 2014; Cervancia et al., 2016), and South Korea in 2019 (Mohamadzade Namin et al., 2019). In China, SHBs were first found to infest Eastern honeybee colonies in 2017 (Zhao et al., 2020). Using a fragment of mitochondrial DNA, the SHBs in China and the Philippines formed a cluster that was distant from beetles collected in other areas, leaving an unresolved invasion source (Liu et al., 2021a). In this study, we used single nucleotide polymorphisms (SNPs) at the genome level to reconstruct the invasion routes of SHBs in China based on a phylogenetic analysis. In addition, the characteristically expressed genes were distinguished between larvae and adults. The number of SNPs in the highly expressed genes was further quantified and compared with the number of SNPs in the remaining genes to infer gene selection.

## Materials and Methods

### Sample Collection

The small hive beetle was first observed to parasitize honeybee colonies in south China in 2017. In July 2019, 23 SHBs, including 8 larvae and 15 adults, were randomly collected from five collapsed honeybee (*Apis cerana*) colonies in the coastal city of Haikou. The SHBs were stored in a −80°C freezer.

### RNA Extraction and Gene Expression Analysis

Out of 23 SHBs, 15 beetles were used for RNA extraction, including 8 larvae and 7 adults. Total RNA was extracted from individual beetle using TRIzol. The RNAseq libraries were prepared for each beetle individually and sequenced on Illumina HiSeq 2000. In total, 15 libraries were sequenced. The quality of RNA-seq reads was controlled using Fastp with default parameters (Chen et al., 2018). The reads were aligned to the SBH genome (GCA_001937115.1) using Hisat2 with default parameters (Kim et al., 2015; Evans et al., 2018). The output files were compressed, sorted, and indexed using Samtools (Li et al., 2009). The variance of replicates was used to calculate significantly regulated genes with the edgeR package and adjusted for multiple comparisons with FDR (Robinson et al., 2010; R Core Team, 2013). The protein sequences of significantly regulated genes were used to query KEGG databases to retrieve the putative functions and involved pathways (Kanehisa and Goto, 2000).

### DNA Extraction, Variant Calling, and Phylogenetic Analysis

DNA was extracted from 8 adult beetles using DNAzol individually. DNA sequencing libraries were prepared for each beetle and sequenced on Illumina HiSeq 2000. In total, 8 libraries were sequenced. In addition, DNA sequencing reads of 48 SHBs were obtained from NCBI for phylogenetic analysis, including samples from South Africa (*N* = 12), United States (*N* = 9), Tanzania (*N* = 6), Burkina Faso (*N* = 12), and Liberia (*N* = 9). Low-quality reads were filtered using Fastp with default parameters (Chen et al., 2018). DNA reads were then mapped to the SBH genome (GCA_001937115.1) using BWA with default parameters (Li and Durbin, 2009). The variants were called using the Picard-GATK-SNPEFF pipeline (Van der Auwera et al., 2013). Then the SNPs of 57 samples were integrated to generate a single gVCF file by CombineGVCFs. The high-quality variants were extracted using GenotypeGVCF and SelectVariants functions in GATK (McKenna et al., 2010; Christmas et al., 2021). The output VCF files were converted to PHYLIP files for genetic phylogenetic analysis using VCF2PHYLIP (Ortiz, 2019). A maximum-likelihood tree was constructed using IQTree with 1000 bootstrap replicates (Nguyen et al., 2015). As the host, the honeybee (*Apis mellifera*) was used as an outgroup to root the tree. The fixation index *F*_*ST*_ was pairwise calculated among the beetles collected in Africa, the United States, and China using PoPoolation2 (Kofler et al., 2011). The distribution of significantly regulated genes in adults and larvae was compared using Pearson’s Chi-squared test, R (R Core Team, 2013). The number of SNPs per gene was compared using a T-test, R. To infer the population structure of the studied beetles, principal component analysis (PCA) was performed using Plink, and the ancestry was estimated using Admixture (Purcell et al., 2007; Zhou et al., 2011).

## Results and Discussion

### Phylogenetic Analysis Suggests Burkina Faso as the Original Source

**FIGURE 1:**
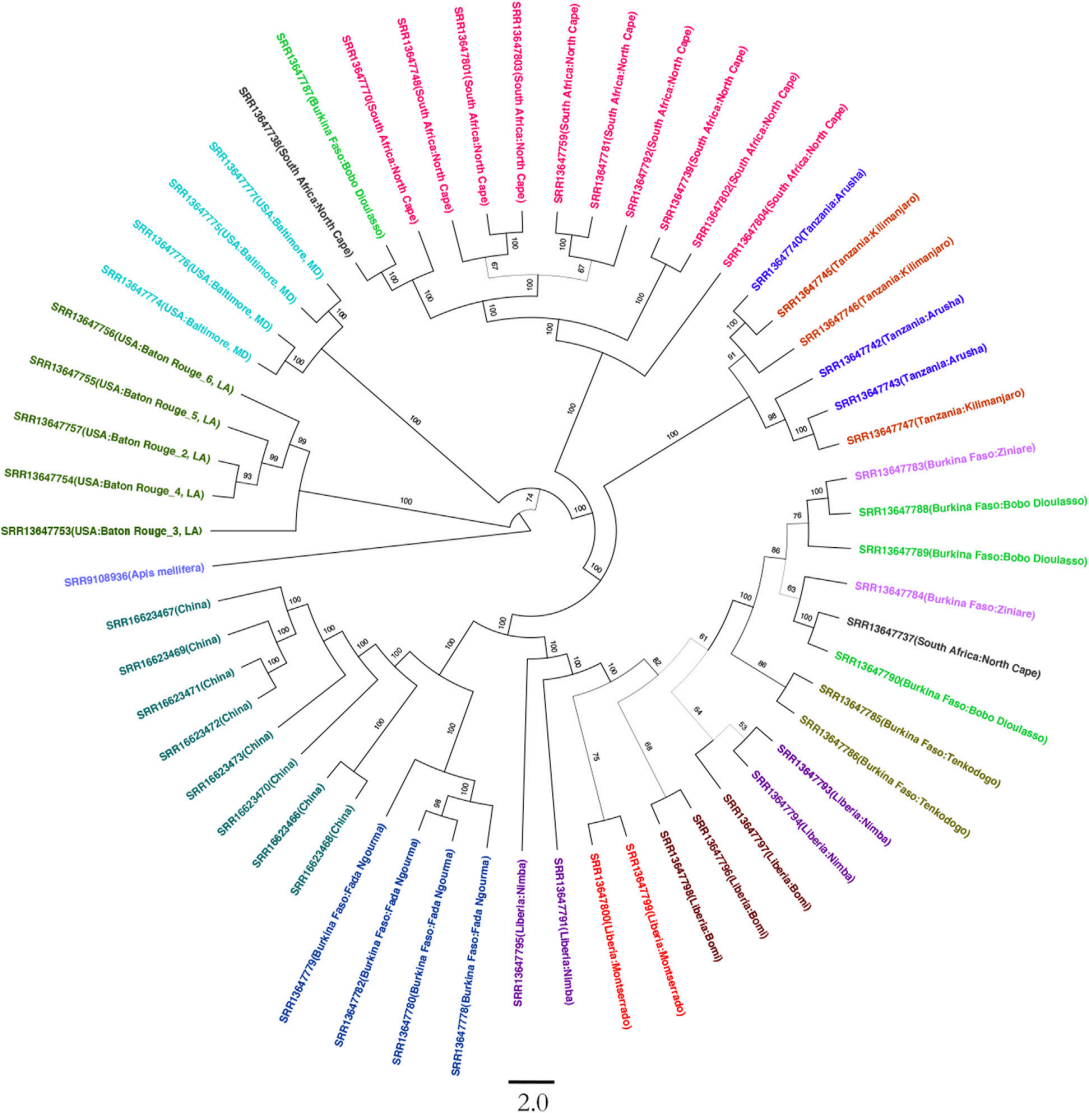
Phylogenetic tree of 56 small hive beetles crossing native and novel regions. The tree was built on 4,541,776 SNPs along the genome. The beetles collected from the same region are highlighted with color. The beetles in the United States were clustered into two clusters. The beetles collected in China formed the deepest branch, which was closely related to those in Burkina Faso. The value on the branch indicates the bootstrap confidence. Honeybee was used as an outgroup to root the tree. The scale bar for branch length is shown at the bottom.

**FIGURE 2:**
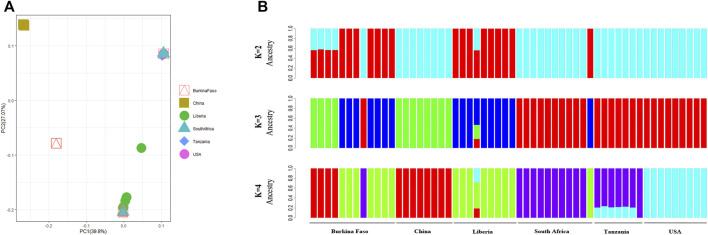
Structure analysis of the beetles. **(A)** PCA plot of the studied beetles. The beetles in China were distant from the ones in native and established regions. **(B)** Ancestral population inference of the studied beetles. The beetles in China may share an ancestor with the ones in Burkina Faso.

On average, 54,358,906 paired reads (151 bp per read) were aligned to the SHB genome, and 5,400,117 SNPs were called in an individual beetle collected in China. After normalizing SHBs collected from Africa and America, 4,541,776 SNPs were identified in all samples, which were used for the phylogenetic analysis. The beetles collected from the United States were clustered into two groups, which formed the earliest branch of studied beetles (Figure 1). The results supported the previous studies that two haplotypes were formed due to multiple intrusions in the United States (Evans et al., 2003; Lounsberry et al., 2010). In addition, the early branch also indicates that the multiple intrusion routes and transporting pollination activities facilitated novel genotypes in the United States, leading to differentiated haplotypes. Indeed, we found that the beetles in the United States showed the highest fixation index with the ones in China compared with those in Africa (Table 1). In our study, the beetles were primarily clustered per country, which reflects that geographic proximity and substantial dispersal of beetles were not detected within their native region. However, occasional intrusions from Burkina Faso to the neighboring countries were indicated, which might be due to human-mediated transportation (Idrissou et al., 2019; Liu et al., 2021b). The PCA recapitulated the occasional dispersal of beetles from Burkina Faso to other regions (Figure 2). In our previous study, the SHBs in China were closely related to the ones in the Philippines based on a fragment of mitochondrial DNA, suggesting that the beetles that invaded the two countries were from the same source (Liu et al., 2021a). However, those beetles cannot be clustered with the ones in their native region. In the PCA plot, the beetles in China were distant from those of other regions. In the phylogenetic tree, the beetles in China and the ones in Burkina Faso formed a cluster, suggesting that the SHBs that invaded China shared a common ancestor with those in Burkina Faso. However, it does not necessarily indicate that SHBs in China were directly imported from Burkina Faso. The ancestry inference analysis also supported the close relationship between the beetles in China and those in Burkina Faso (Figure 2). In Asia, the beetles were also reported to invade South Korea. Using mitochondrial DNA, it was found that the beetles in Korea likely originated from the United States (Mohamadzade Namin et al., 2019). It seems clear that SHBs invaded Asia through multiple invasion paths.

**TABLE 1:** Pairwise fixation index *F*_*ST*_ of the three beetle sources along the genome (mean ± SE). The beetles collected from China and the United States showed the highest divergence.

Beetle Source	China	United States
Africa	0.1432 ± 0.0141	0.062 ± 0.0078
United States	0.2308 ± 0.0225	—

### Gene Expression of Larvae and Adults

**FIGURE 3:**
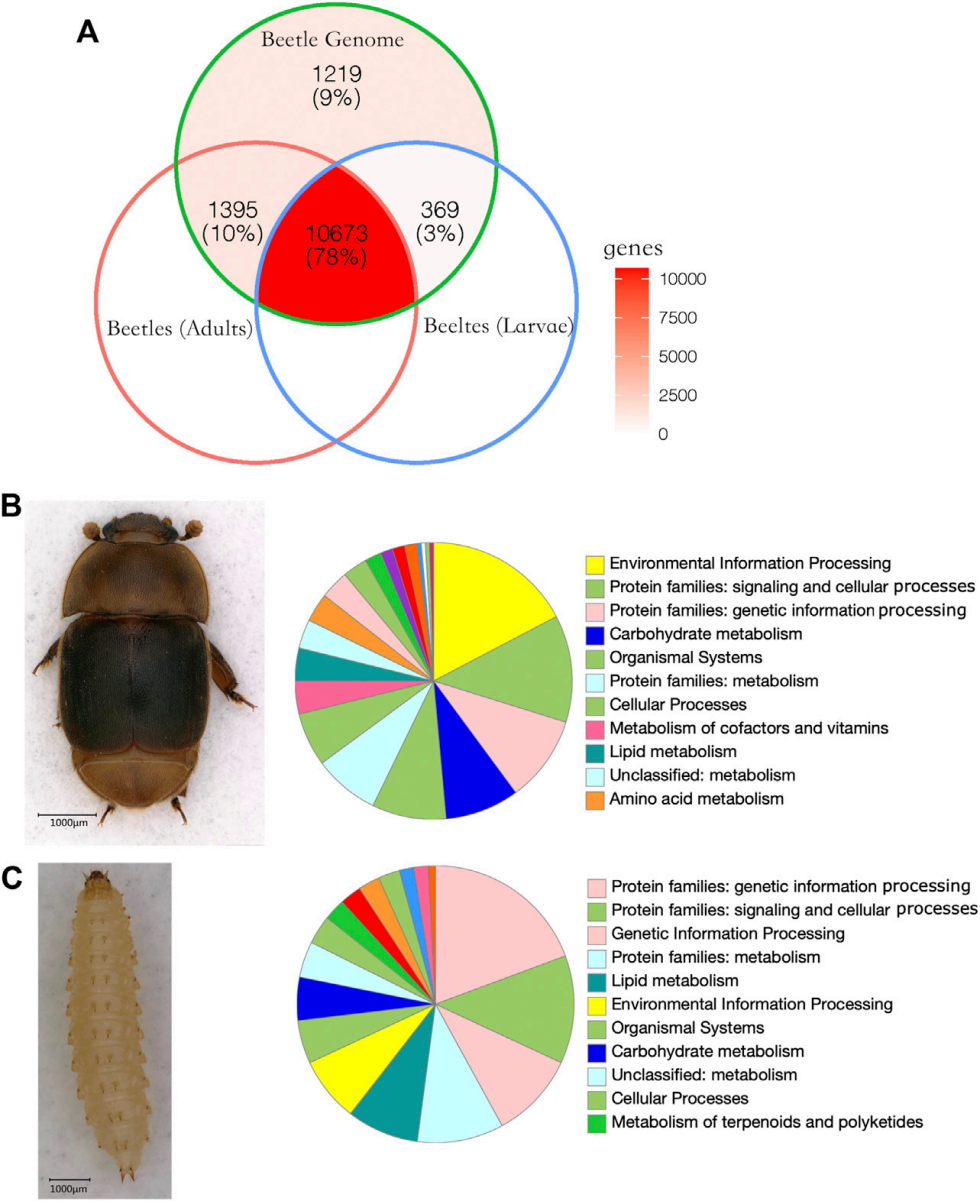
Gene expression of small hive beetles. **(A)** Venn diagram of expressed genes in SHB adults and larvae. In the beetle genome, 13,656 protein-coding genes were annotated. Overall, 78% genes were expressed in both adults and larvae. **(B)** Significantly upregulated genes in adults. In total, 460 genes were significantly upregulated in adults compared with larvae SHBs, and the genes were enriched in environmental information processing. **(C)** Significantly upregulated genes in larvae. Overall, 203 genes were significantly upregulated in larvae compared with adults and the genes associated with translation, replication, and repair showed a high relative abundance compared with adults.

On average, 29,249,729 paired reads (151 bp per read) were aligned to SHBs. Out of 13,656 annotated protein-coding genes, 12,068 genes were detected in adults, and 11,042 genes were detected in larvae (Figure 3). Among the expressed genes, 203 genes were significantly upregulated in larvae, and 460 genes were significantly upregulated in adults, which significantly deviated from random (Pearson’s Chi-squared test, df = 1, *p* < 0.001). By quantifying the number of SNPs in each gene, we found that the 663 overexpressed genes in adults and larvae showed a significantly higher number of SNPs than the remaining genes (*t*-test, *p* < 0.05). Using the Kyoto Encyclopedia of Genes analysis, the distribution of genes in six main pathways was significantly different between adults and larvae (Pearson’s Chi-squared test, df = 5, *p* < 0.001). Among the six main pathways, metabolism was associated with the highest number of genes in both adults (31.0%) and larvae (40.1%). The larvae showed a higher number of upregulated genes associated with translation, replication, and repair than the adults. In the white-striped longhorn beetle, more genes were upregulated in adults than in larvae, and the antennae showed tissue-specific expressed olfactory genes (Yang et al., 2018). Similarly, in the coffee berry borer beetle, more genes were significantly upregulated in adults than in larvae. As expected, higher expression levels of larval-specific cuticle-binding proteins and chitinases were found in larvae (Noriega et al., 2019). In another pest beetle, *Tribolium castaneum*, a higher number of upregulated genes were found in larvae than in adults. Large numbers of ribosomal proteins were included, indicative of protein production variation between the life stages (Perkin and Oppert, 2019). In our data, the relative abundance of genes involved in genetic information processing was two-fold higher in larvae than in adults, which indicates the fast growth at this stage as supported by highly expressed metabolic genes.

## Data Availability

The datasets presented in this study can be found in online repositories. The names of the repository/repositories and accession number(s) are as follows: NCBI PRJNA782806 and PRJNA776042.
